# Food security and emerging infectious disease: risk assessment and risk management

**DOI:** 10.1098/rsos.211687

**Published:** 2022-02-16

**Authors:** Valeria Trivellone, Eric P. Hoberg, Walter A. Boeger, Daniel R. Brooks

**Affiliations:** ^1^ Illinois Natural History Survey, Prairie Research Institute, University of Illinois at Urbana Champaign, 1816 South Oak Street, Champaign, IL 61820, USA; ^2^ Department of Pathobiological Sciences, School of Veterinary Medicine, University of Wisconsin-Madison, WI 53716, USA; ^3^ Museum of Southwestern Biology, Department of Biology, University of New Mexico, Albuquerque, NM 87131, USA; ^4^ Biological Interactions, Universidade Federal do Paraná, Cx Postal 19073, Curitiba, Brazil; ^5^ Department of Ecology and Evolutionary Biology, University of Toronto (emeritus), Toronto, ON, Canada; ^6^ Harold W. Manter Laboratory of Parasitology, University of Nebraska-Lincoln, NE 68588-0514, USA; ^7^ Institute for Evolution, Centre for Ecological Research, Karolina ut 29, Budapest, Hungary H-1113

**Keywords:** climate change, habitat interfaces, pathogen spillover, trade, Stockholm paradigm, DAMA protocol

## Abstract

Climate change, emerging infectious diseases (EIDs) and food security create a dangerous nexus. Habitat interfaces, assumed to be efficient buffers, are being disrupted by human activities which in turn accelerate the movement of pathogens. EIDs threaten directly and indirectly availability and access to nutritious food, affecting global security and human health. In the next 70 years, food-secure and food-insecure countries will face EIDs driving increasingly unsustainable costs of production, predicted to exceed national and global gross domestic products. Our modern challenge is to transform this business as usual and embrace an alternative vision of the biosphere formalized in the Stockholm paradigm (SP). First, a pathogen-centric focus shifts our vision of *risk space*, determining how pathogens circulate in realized and potential fitness space. *Risk space* and pathogen exchange are always heightened at habitat interfaces. Second, apply the document-assess-monitor-act (DAMA) protocol developing strategic data for EID risk, to be translated, synthesized and broadcast as actionable information. *Risk management* is realized through targeted interventions focused around information exchanged among a community of scientists, policy practitioners of food and public health security and local populations. Ultimately, SP and DAMA protect human rights, supporting food security, access to nutritious food, health interventions and environmental integrity.

## 1. Introduction

Emerging infectious diseases (EIDs), affecting animals—including humans—and plants, are an exigency in a time of global climate change [1,2]. Food production systems are especially at risk (e.g. [3–5]). Climate change and anthropogenic forcing drive species movement, leading to ecological mixing through altered habitat interfaces (i.e. boundary zones, of varying extent, that are the nexus for pathogen exchange across managed (anthropogenic) and wildland habitats). Landscape interfaces allow pathogens to come into contact with susceptible but previously unexposed hosts, producing EIDs [1,6–11]. Food-safety standards have been expected to prevent known and re-emerging diseases, but have failed to anticipate new EIDs impacting consumers along the global value chain (e.g. [12]). Food security, however, is considerably broader encompassing global food supply chains for domestic animals, crop/plant-based resources and managed systems in freshwater and marine environments where access and availability must be guaranteed [13]. Humans expand the risk space for food insecurity through changing land use and by behaviours that add to climate forcing [11,14–16]). Technological advances are not resolving growing inequities and unsustainability in global food production and distribution [17].

EIDs limit the production and distribution of food, contributing directly and indirectly to global food insecurity [13,18–20]. Regional populations in food-insecure countries depend directly and indirectly on agriculture. The current Global Report on Food Crises [21] states that approximately 103.2 million people in 10 countries face food crises. EIDs disrupt supply chains and contribute to market losses that drive increasing prices further limiting access, especially for countries experiencing prolonged crisis [17]. A threat network challenges the sustainability and security of agricultural and food systems ([1,13] and references therein). For example, nearly two-thirds of the population of Yemen rely on agriculture and livestock for subsistence. In this restricted geographical microcosm, an exemplar for many vulnerable countries, air- and vector-borne animal infectious diseases (i.e. *Capripoxvirus* and *Phlebovirus*), water-borne bacterial pathogens (i.e. *Vibrio cholerae*, cholera) and new severe acute respiratory syndromes (Coronaviridae) in humans further impact food security. Despite situations that demand novel approaches, containment strategies in Yemen and other countries rely mainly on vaccination [22,23].

Only five countries have a high performance in terms of food security indices [24]. The poorest countries are expected to see net imports rise more than 28% by 2030 [25].

**Figure 1.**
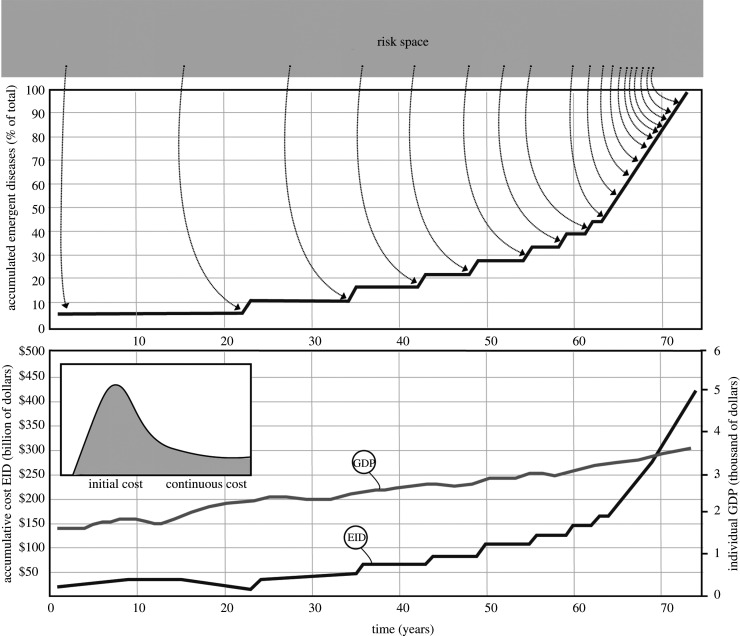
Simulated cumulative costs (*y* axis on the left of lower graphic) associated with the emergence of a new disease/pest in food production (e.g. aquaculture, agriculture) (dark-grey curve) in a food-secure country, United States (US). Predicted growth of per capita GDP (gross domestic product, *y* axis on the right) for the next 80 years (light-grey curve, graph at the bottom). Data retrieved from the World Bank database [26]. In the simulation, the rate of emergence increases exponentially during a period of time over 80 years. In the first 20 years, costs associated with reactive responses (e.g. control measures) to the new EID decrease slightly over time (insert in lower graphic). Once the pathogen associated with the EID is introduced in the production system the cost is internalized as a fixed cost that becomes cumulative. In the next 70 years, overall costs increase exponentially and exceed the curve of individual GDP. The graph at the top denotes cumulative and accelerating increases over time with the sequential emergence of new diseases originating from the *risk space*.

Increasing costs of integrated management, along with production losses due to infectious disease in crops and livestock are cumulative and unsustainable (figure 1). Direct costs associated with farming strategies cause economic losses in downstream industries (processors, shippers, marketers) (e.g. [20]). Indirect costs have been viewed as business costs passed along to consumers as higher prices and lower supply. These include ‘ghost’ impacts on economies and health, which may triple total costs (e.g. [27]). Annual yield losses of only 0.01 and 0.1% due to the fungal disease wheat karnal bunt causes millions of dollars of costs associated with quarantine measures and trading restrictions [28]. Import restrictions on wheat imposed by the USA after 1996 resulted in revenue losses of $250 million [29]. Indirect costs of animal diseases such as avian influenza [30] maybe 5 to 10 times more than direct costs. Simulations for a hypothetical outbreak of African swine fever (ASF) in Denmark, important pork meat exporters, showed that indirect costs maybe 29 times larger than direct costs [31].

Humanity has tried to eradicate pathogens and diseases in food production or at least mitigate their impact for millennia. New management practices, pesticides or vaccines, genetic manipulation and selection for resistance are part of today's business as usual which emphatically is not working.

We are in a socio-economic death spiral and we need to change how we view the biosphere and pathogens if we are to escape and survive [1,7,13]. This begins with a new paradigm of pathogen–host evolution. Business as usual follows a model predicting that disease emergence should be rare. And colonizing new hosts requires novel genetic mutations for exploiting those new hosts. Shifts among potential hosts are rare because pathogen variation and geographical and host ranges are limited by ecological specialization resulting from coevolutionary interactions [32,33]. Inconsistencies in the standard model, especially high numbers of EIDs, produced the parasite paradox [32].

The Stockholm paradigm (SP) resolves the parasite paradox and this fundamental confusion over apparent generalists and specialists [1,34–39]. We have identified generalist pathogens historically as species associated with a broad range of hosts, but this is misleading. The explanation for pathogens inhabiting an array of hosts as specialists is that specialized host resources are phylogenetically conservative and widespread. Host range at any particular point in time then reflects the interaction of this capacity for resource use, and changing opportunity, which may either broaden or restrict actual host range in space and time [1,40]. A wide range of pathogens may have similar transmission dynamics and microhabitat preferences within hosts. There may be many susceptible but unexposed hosts needing only a change in geographical distribution or trophic structure to acquire a new pathogen [32]. Throughout evolutionary history, climate perturbations allow pathogens to oscillate between exploring fitness space that is inherently sloppy [1,32,36], encountering a diverse assemblage of susceptible hosts and exploiting hosts during periods of unstable environmental conditions (e.g. [1,7,32,34,36] and references therein). New pathogen–host associations resulting from changing opportunity set the stage for *subsequent* emergence of genetic innovations. Empirical evidence from deep- and shallow-time phylogenetic studies show clearly that environmental change and associated geographical expansion are correlated with host range expansion leading to emerging diseases (e.g. [1,34,37,39]). A new modelling platform [34,37,39,41] shows the feasibility of the SP dynamics to produce today's disease emergences.

**Figure 2.**
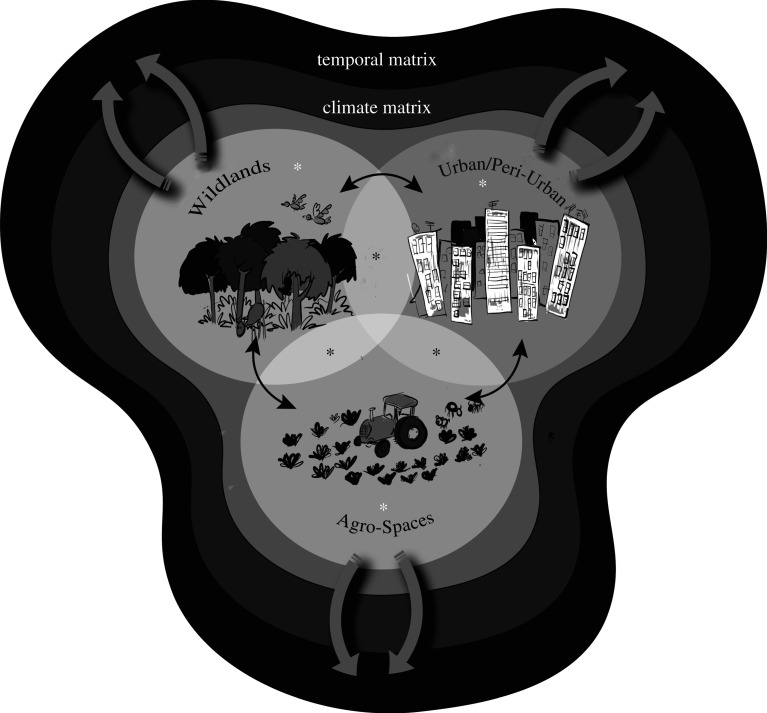
The three major landscapes (Wildlands, Urban/Peri-Urban, Agro-Spaces) and habitat interfaces (black stars) representing higher risk spaces for EIDs to occur (bidirectional black arrows). Connectivity within and among the landscapes is dynamic (temporal matrix) and includes passive (e.g. climate responses) and active pathways (e.g. globalization, land use). In a proactive capacity, interventions are most appropriate and effective within landscapes (white asterisks) and among interfaces (black asterisks), which we define as *intervention space*, in order to prevent triggers for pathogen expansion.

The margins of urban/peri-urban, wildland and agroscape environments represent the habitat interfaces where EIDs are most likely to occur and re-occur (figure 2). Connectivity within that space is dynamic with pathways influencing pathogen and disease distribution. As Audy [42] noted more than half a century ago, pathogen distribution is not homogeneous and the geographical and host range of a pathogen always exceeds that of disease caused by it. This has been characterized as a minefield in which EIDs are evolutionary accidents waiting to happen [1,3,43].

## 2. Risk space

Increasing food security with respect to EIDs requires that we visualize a global arena of expanding *risk space*. Assessing that space requires extensive knowledge of the interfaces between wildlands and managed landscapes (figure 2, black stars), transmission dynamics and microhabitat preferences for pathogens and the range of potential hosts. This is best understood by taking the pathogen's point of view and not the host's [1,36,44–46].

Our traditional host-centric focus has kept us from being able to assign risk, given the expectation that pathogen emergence is rare and unpredictable. We focused on diseased hosts neglecting the true source of infectious disease, asymptomatic reservoirs and the main actor, pathogens. The SP combines climate, environmental and diversity parameters to determine *fundamental pathogen fitness space* (the entire realm within which any pathogen could survive). Within that space, interconnectivity and isolation, both temporal and spatial, determine *realized pathogen fitness space*. *Risk space* is maximized along the habitat interfaces and is widely interconnected, becoming more so as a result of global climate change and anthropogenic forcing (e.g. [13,34,37,38,47]).

Cereals provide about half of the world's caloric intake [47] and pathogens in the *Pucciania graminis* complex of rust fungi cause serious diseases of staple crops. Although *P. graminis* may infect 365 species across 54 genera, many of them not food plants, it is still considered a host-specialist [48]. Despite efforts to breed resistant wheat cultivars, the stem rust of wheat (*P. graminis* f. sp*. tritici*) poses a significant threat, expanding its geographical range and re-emerging in places where it was thought to have been eradicated [49]. Rust fungi spores are dispersed through the air. The overwintering region is expanding northward from the original tropical distribution [50], accelerated anthropogenically [51]. The risk of increased virulence and persistence depends on interfaces between crop fields and habitats that may harbour alternative hosts (figure 2, black stars). Many food-secure countries, e.g. in Europe, have all the predisposing factors for a rapid re-establishment of the pathogen. Oversimplified projections do not allow accurate estimation of the risk, and other factors need to be included in the modelling efforts (such as climate, land use) as suggested [50,52,53].

## 3. Risk management

*Risk management* for EIDs requires actionable information *before a crisis appears*. Strategic protocols obtaining such information have been largely missing in PREDICT and One Health proposals [8,54–59]. Actionable information obtained *in advance of disease emergence* can only be achieved if we think that we can prevent EIDs or at least mitigate their impact greatly. We understand that prevention is a cost-effective alternative to crisis response when talking about metabolic disease (e.g. heart disease, diabetes), but have not applied those principles to infectious disease. The SP suggests that the cost-effectiveness of prevention is possible for EIDs if we find them before they find us, or our food [1,13,60]. Because they are phylogenetically conservative, the specialized traits that allow pathogens to capitalize on new host opportunities, making EID *risk space* large, also make their behaviour in novel settings predictable.

The DAMA (document-assess-monitor-act) protocol [1,6] stems from the SP.

(1) Document—explore, find and identify the diverse assemblages of potentially pathogenic micro- and macro-parasites living in the interfaces where they can be encountered by susceptible humans, crops and livestock (figure 2, black stars), and document their circulation and exchange among domestic, semi-domestic and wild animals and plants between wildlands and agro-systems (figure 2, black arrows). Make use of local knowledge in areas being sampled. Provide the following information: (i) what known pathogens occur in a place, (ii) where else do they occur, (iii) what are their reservoirs, (iv) what is their prevalence/distribution in populations of hosts, and (v) what environmental factors enhance their survival, and where do those conditions occur? [37]. ‘High-risk’ pathogen diversity remains poorly documented, especially within wildlife landscapes (figure 2, white star) (e.g. [61,62]). Sampling from biorepositories in conjunction with strategic on-the-ground and real-time sampling at expanding interfaces can provide a nuanced picture of the biosphere and place pathogens in a broader global context [61]. Store specimens of all discovered pathogens and their hosts in properly curated archival collections which become critical baselines. Store the information digitally and link it to the interfaces (figure 2).
(2) Assess—determine the suite of microbes of special concern that should be monitored closely. This is a two-part process. The first step is phylogenetic triage: place each discovered microbe in a phylogenetic context and ask is it a known pathogen? If so, report it to the relevant health authorities. Are they close relatives of known pathogens that are not known to cause disease but might? If so, monitor. Are they related to non-pathogens. If so, ignore but archive for future reference. Phylogenetic triage can also be used to anticipate potential host range and likely transmission dynamics for pathogens related to species known to cause disease [1,45]. The second step involves using the SP modelling platform [34,39,41] to assess the genetic status of potential pathogens of concern. Contemporaneous data need to be linked with information about past conditions using archival resources in natural history museums and biodiversity repositories (e.g. [57,61,63]) to predict additional places where the pathogen might occur or successfully establish itself [34,37].
(3) Monitor—regularly re-sample potential or known pathogens of interest in areas where they have been discovered and search for them in areas predicted to be suitable for them (vulnerable areas, such as areas of new introduction). Re-assess using the modelling platform to alert us to changes in the pathogen population being monitored. Monitoring and surveillance generate actionable information about expanding *risk space* (e.g. the extent of anthropogenic and environmental disruption across habitat interfaces).
(4) Act—those responsible for disease-related food and public health security need to formulate action plans, based on the implications of the SP, adapted to specific socio-cultural-economic situations. Intervention space includes within (figure 2, white stars) and between (figure 2, black stars) landscapes; critical space for *risk management* are the interfaces. Most importantly, natural history collections must be recognized as the critical hubs for data gathering. The synthesis requires dedicated databases and personnel trained to translate data into actionable information. Resources and expertise must be maintained, expanded, and used in action plans. A global action is realized when shared comprehensive DAMA programmes within and specific to each country are established and interconnected. Robust pathways for sharing information and resources are critical with recognition of how globalization influences pathogen distribution in rapid timeframes. The programmes will need to integrate global climate change, global trade and global travel as transboundary factors. Only then can we take advantage of the cost-effectiveness of prevention over crisis response [1,6].

**Figure 3.**
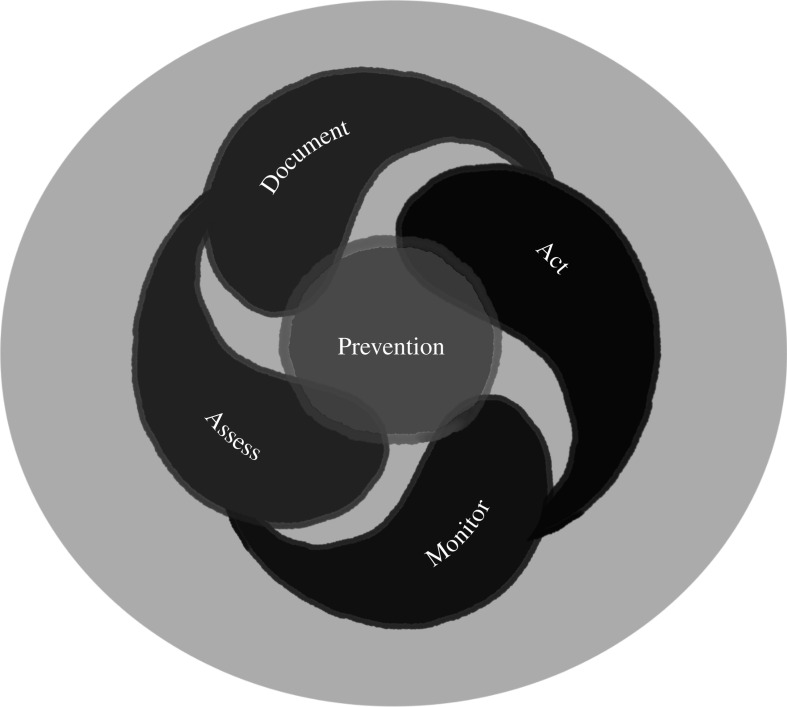
The DAMA (document-assess-monitor-act) protocol scheme. Food security is improved by taking into account risk space of emerging infectious diseases, and through an informed management that stems from linking data and information generated by DAMA.

Information must circulate easily and DAMA concatenates pieces of translated data into effective action. In the Information Age, some data are not easily accessible [2] preventing us from linking all the DAMA elements; these barriers must be eliminated. DAMA initiatives will work best when information is shared freely among all concerned parties. This is *anticipate to prevent* (figure 3) (e.g. [1,64]), producing specific time-sensitive recommendations that can avert outbreaks of diseases.

## 4. Conclusion

Human rights include healthy and safe living conditions, food and water security, nutritious food, adequate health care and environmental integrity. Mitigating EIDs at an affordable cost [4,19,65,66] (figure 3) should be a priority that can be achieved by applying the scientific principles of the SP through its policy extension, DAMA [1,6,13,60]. Reducing the disease costs to food production will lower costs, allowing people better access to food with higher nutritional content.

The link between EIDs and global climate change mediated by interfaces for transmission within and between wildlands and managed landscapes makes this a priority for efforts to provide a sustainable future for humanity.

## Data accessibility

This article has no associated data.
